# Metabolic Determinants in Cardiomyocyte Function and Heart Regenerative Strategies

**DOI:** 10.3390/metabo12060500

**Published:** 2022-05-31

**Authors:** Magda Correia, Francisco Santos, Rita da Silva Ferreira, Rita Ferreira, Bruno Bernardes de Jesus, Sandrina Nóbrega-Pereira

**Affiliations:** 1 Department of Medical Sciences, Institute of Biomedicine—iBiMED, University of Aveiro, 3810-193 Aveiro, Portugal; tcorreiapm@ua.pt (M.C.); franciscojfsantos@ua.pt (F.S.); ritasferreira@live.ua.pt (R.d.S.F.); 2 Associated Laboratory for Green Chemistry—LAQV, Department of Chemistry, University of Aveiro, 3810-193 Aveiro, Portugal

**Keywords:** cell reprogramming, pluripotency, metabolism, nutrient signaling, cardiomyocytes, cardiac regeneration, mitochondria

## Abstract

Heart disease is the leading cause of mortality in developed countries. The associated pathology is characterized by a loss of cardiomyocytes that leads, eventually, to heart failure. In this context, several cardiac regenerative strategies have been developed, but they still lack clinical effectiveness. The mammalian neonatal heart is capable of substantial regeneration following injury, but this capacity is lost at postnatal stages when cardiomyocytes become terminally differentiated and transit to the fetal metabolic switch. Cardiomyocytes are metabolically versatile cells capable of using an array of fuel sources, and the metabolism of cardiomyocytes suffers extended reprogramming after injury. Apart from energetic sources, metabolites are emerging regulators of epigenetic programs driving cell pluripotency and differentiation. Thus, understanding the metabolic determinants that regulate cardiomyocyte maturation and function is key for unlocking future metabolic interventions for cardiac regeneration. In this review, we will discuss the emerging role of metabolism and nutrient signaling in cardiomyocyte function and repair, as well as whether exploiting this axis could potentiate current cellular regenerative strategies for the mammalian heart.

## 1. Introduction

**Figure 1:**
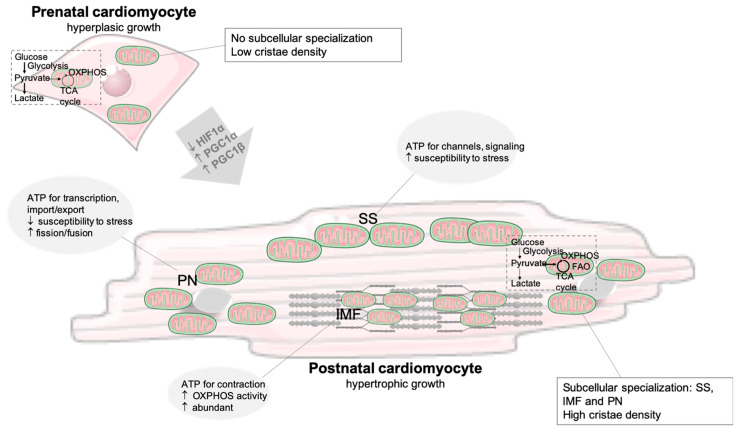
Connection between cardiomyocyte development and mitochondria usage and maturation, highlighting mitochondria specialization according to the spacial arrangement in postnatal cardiomyocytes. After birth, a bioenergetic shift from glucose-driven anaerobic glycolysis to the oxidation of fuels in the mitochondria occurs. Mitochondria increase in size and density, and cristae become denser. The upregulation of PGC coactivators and the downregulation of HIF1α regulate mitochondria biogenesis. Abbreviations: FAO, fatty acid oxidation; HIF, hypoxia-inducible factor; IMF, intermyofibrillar; OXPHOS, oxidative phosphorylation; PGC, peroxisome proliferator-activated receptor-gamma coactivator; PN, perinuclear mitochondria; SS, subsarcolemmal; TCA, tricarboxylic acid cycle; up arrow: increase; low arrow: decrease.

Cardiovascular diseases are the leading cause of morbidity and mortality worldwide [1]. This is largely due to the inability of the adult mammalian heart to replenish the lost myocardial tissue following injury, which results in the progressive weakening of the heart muscle and the development of heart failure [2]. Although, currently, there is a large range of pharmaceutical drugs and surgical options that prevent further deterioration or restore function to the failing heart, the only long-term solution for end-stage heart failure is heart transplantation [3]. Several regenerative strategies to repair the injured heart and improve heart function have been pursued, including post-injury activation of cardiomyocyte proliferation, recruitment of cardiac progenitor cells, delivery of embryonic stem cells (ESCs) or induced pluripotent stem cells (iPSCs)-derived cardiomyocytes, and direct reprogramming of somatic cells into induced cardiomyocytes (for a revision, see [3,4]). Besides holding great promise, most cardiac regenerative strategies still lack effective clinical outcomes [3,4]. Thus, understanding the molecular mechanisms and players governing cardiomyocyte function is warranted for improving the efficiency of cardiac regenerative strategies and the patient’s welfare. Following an injury, the capacity for regeneration of the adult mammalian heart is limited, with an estimation of only 1% De Novo cardiomyogenesis per year [5], while the neonatal heart is capable of substantial regeneration, but this capacity is lost by postnatal day (P) 7 [6]. The transition from neonatal to adult heart is accompanied by profound cellular and metabolic changes. Interestingly, this loss of proliferative potential is accompanied by a bioenergetic shift in cardiomyocytes, from glucose-driven anaerobic glycolysis to oxygen-dependent oxidative phosphorylation of pyruvate and fatty acids (FAs), in the mitochondria [7,8]: the so-called fetal metabolic switch (Figure 1). The healthy heart is the most energetically demanding and metabolically versatile organ [9,10,11], and upon injury, metabolic reprogramming compensates for impaired fuel utilization and loss of mitochondrial functionality in the diseased heart [12]. Apart from energetic sources, metabolites, including those that are mitochondria-derived, are key regulators of gene expression programs by acting as essential substrates or cofactors for chromatin-modifying enzymes [13] and regulating histone landscape transitions that drive adult stem cell regeneration [14,15,16]. Understanding the impact of systemic metabolism and nutrient signaling in cardiomyocytes’ self-renewal, differentiation, and maturation may bring new opportunities for cardiac regeneration.

In this review, we highlight major studies of metabolism and nutrient signaling in cardiomyocyte function, including insights into metabolic reprogramming during development and in the injured heart. We also discuss key metabolic determinants and manipulations that drive cellular pluripotency and cardiac differentiation, which have been shown to improve current heart regenerative strategies.

## 2. Metabolism in the Adult Heart: Mitochondria and Fuels

The healthy adult heart is highly reliant on fatty acid oxidation (FAO, 50–70%) for ATP generation, while the remaining percentage is mostly assured by glucose oxidation. Ketone bodies, mainly β-hydroxybutyrate (βOHB), are the third main supplier of the tricarboxylic acid (TCA) cycle (10–15%), whereas amino acids only contribute 1–2% for energetic purposes [17]. The heart can use a variety of amino acids as fuel sources, including branched-chain amino acids (BCAAs), glutamate, cysteine, histidine, and lysine [18]. The contribution of these substrates as energetic suppliers highly depends on their circulating levels, hormonal status, and cardiac workload [17]. Mitochondria are the ultimate fate of fatty acyl-CoA and pyruvate, products of lipolysis or free fatty acids (FFAs) uptake and glycolysis, respectively, and they are the place in the cell where ketone bodies are converted into acetyl-CoA and amino acids undergo transamination [17,19].

Mitochondria occupy around 30% of cardiomyocytes’ volume to accommodate the high ATP demands that drive cardiac contraction/relaxation (around 90%) and ionic homeostasis. In fact, cardiomyocytes, out of all the cell types, exhibit the highest mitochondrial density, which reflect the key role of this organelle in the cardiac remodeling, induced by several pathophysiological conditions [12,19,20,21]. The organization of mitochondria within cardiomyocytes is determined to support heart function [22]. In adult cardiomyocytes, mitochondria are spatially arranged in three different subpopulations: intermyofibrillar (IMF), subsarcolemmal (SS), and perinuclear (PN) mitochondria (Figure 1). IMF mitochondria, the most abundant subpopulation, energetically support the contractile activity of cardiomyocytes, whereas the SS subpopulation provides ATP for membrane activities (channel and signaling functions), and the PN mitochondria provide energy for transcription and import/export processes [19,23,24].

**Figure 2:**
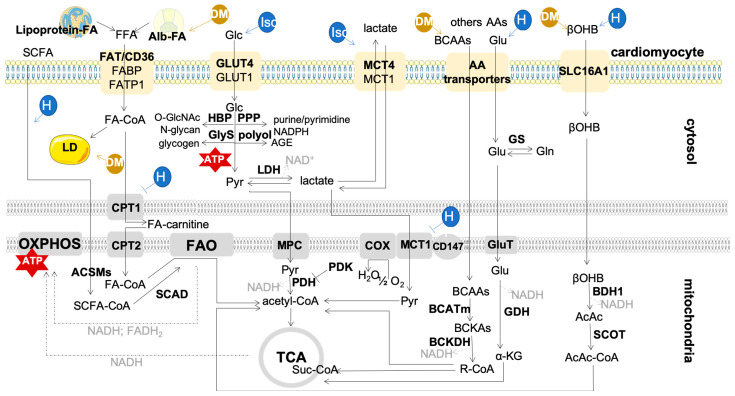
Energetic metabolism of cardiomyocytes. FFAs (free fatty acids), the main energetic fuel, are converted into long-chain acyl-CoA esters by fatty acyl CoA synthetase, and then, into long-chain acylcarnitine by CPT1, the rate limiting enzyme of long-chain FAO. Inside the mitochondria, long-chain acylcarnitine is converted back to long-chain acyl-CoA by CPT2, and then, several isoforms of acyl-CoA dehydrogenase, enoyl-CoA hydratase, 3-hydroxyacyl CoA dehydrogenase, and 3-ketoacyl CoA thiolase (specific for different chain lengths) mediate the shortening of the fatty acyl moiety, generating acetyl-CoA, FADH2, and NADH. Glc is transported by GLUTs (GLUT4 in adult heart) into cardiomyocytes, and, in the cytosol, Glc is converted into pyruvate through glycolysis. Pyruvate may be oxidized in mitochondria or converted into lactate by LDH in the cytosol. Lactate uptake and secretion is mediated by MCTs (MCT4 in the adult heart). MCT1 makes part of the mtLOC, which is also composed of CD147, LDH, and COX. Lactate may be oxidized in the mitochondria by this complex. Ketones, mostly βOHB, may feed TCA after being metabolized by the enzymes β-hydroxybutyrate dehydrogenase 1 (BDH1, the enzyme that interconverts β-hydroxybutyrate into acetoacetate) and succinyl-CoA:3-ketoacid CoA transferase (SCOT, the rate-limiting enzyme of ketone oxidation). SCFAs (including acetate, propanoate, and butyrate) can cross the mitochondrial membrane by diffusion, and enter FAO through SCAD, after being activated via ACSMs. BCAA and other amino acids, such as Glu, may be used for energetic purposes by feeding the TCA cycle. BCATm and BCKDH catalyze the first two enzymatic steps in BCAA metabolism. The contribution of these metabolic pathways changes in the injured heart. In ischemia (Isc), the reliance on glucose and lactate increases, but when insulin signaling is repressed, FAO is upregulated. During heart failure (HF) glucose oxidation prevails until mitochondria functionality is impaired, which happens at advanced stages of disease. In diabetes mellitus (DM), glucose uptake and oxidation decrease, inducing a higher reliance of cardiac metabolism on lipids for energetic purposes. Abbreviations: α-KG, α-ketoglutarate; βOHB, β-hydroxybutyrate; AA, amino acid; AcAc, acetoacetate; ACSMs, acyl-coenzyme A synthetase medium chain family members; AGE, advanced glycation end products; Alb, albumin; ATP, adenosine-5’-triphosphate; BCAA, branched chain amino acids; BCATm, mitochondrial branched-chain aminotransferase; BCKDH, branched-chain α-ketoacid dehydrogenase complex; BDH1, 3-hydroxybutyrate dehydrogenase 1; CD147, cluster of differentiation 147; COX, cytochrome c oxidase; CPT, carnitine palmitoyl transferase; DM, diabetes mellitus; FA-carnitine, acyl-carnitine; FA-CoA, long-chain acyl-CoA; FABP, fatty acid binding protein; FAO, fatty acid oxidation; FAT/CD36, fatty acid translocase; FATP, fatty acid transport protein; FADH_2_, reduced flavin adenine dinucleotide; GDH, glutamate dehydrogenase; Glc, glucose; Gln, glutamine; Glu, glutamate; GLUT, glucose transporter; GluT, glutamate transporter; GlyS, glycogen synthesis; GS, glutamine synthase; HBP, hexosamine biosynthetic pathway; HF, heart failure; Isc, ischemia; LD, lipid droplet; LDH, lactate dehydrogenase; MCT, monocarboxylate transporter; MPC, mitochondrial pyruvate carrier; NAD^+^, oxidized nicotinamide adenine dinucleotide; NADH, reduced nicotinamide adenine dinucleotide; O-GlcNAc, O-linked β-N-acetylglucosamine; OXPHOS, oxidative phosphorylation; PDH, pyruvate dehydrogenase; PDK, pyruvate dehydrogenase kinase; PPP, pentose phosphate pathway; Pyr, pyruvate; R-CoA, branched-chain acyl CoAs; SCOT, succinyl-CoA: 3-oxoacid-CoA transferase; SCAD, short chain acyl-CoA dehydrogenase; SCFA, short chain fatty acids; SLC16A, solute carrier family 16 member 1; Suc-CoA, succinyl-CoA; TCA, tricarboxylic acid cycle.

Regardless of the cellular location, the major function of mitochondria is to support ATP generation through the OXPHOS process, which is primarily reliant on the reducing equivalents obtained from the oxidation of long-chain fatty acids (LCFAs). Circulating FA (lipoprotein-derived and albumin-derived FAs) are taken up by a family of transporters, including FA translocase (FAT/CD36), plasma membrane fatty acid-binding protein (FABP), and FA transport protein 1 (FATP1), with FAT/CD36 showing greater efficiency [25,26,27] (Figure 2). The translocation of these transporters to the sarcolemma is crucial for FA uptake and oxidation, and it is regulated by contraction, insulin, and AMP-activated protein kinase (AMPK). In fact, changes in the relocation of these transporters have great impact on the use of FA for energetic purposes [26] since the energetic stores (such as triglycerides, TG) are limited, supporting only a few heart beats [19] (Figure 2).

FAO is co-regulated with other metabolic pathways to assure homeostasis when the environmental conditions change. Such reciprocal regulation in the heart is mostly noticeable between FAO and glucose oxidation [26,28]. Glucose, the second main fuel, is translocated into cardiomyocytes through the glucose transporters (GLUTs)—mainly GLUT4 (Figure 2). This glucose transporter resides in intracellular storage vesicles, being translocated to the sarcolemma in response to stimuli, such as insulin, increased workload, and catecholamines [29]. Glucose is converted into pyruvate through glycolysis, and pyruvate is then oxidized in mitochondria. Regarding ATP generation efficiency, glucose oxidation is more cost-effective compared to FAO (2.58 vs. 2.33 P/O ratio) [11]. Glycolysis contributes with a small percentage of ATP (less than 10%), which is mostly used to energetically support ion pumps [30]. However, the role of glycolysis goes beyond the energy supply. This metabolic pathway feeds other pathways with intermediate metabolites, such as glycogen synthesis, hexosamine biosynthetic, polyol, and pentose phosphate pathways that regulate cell proliferation, redox balance, and transcription. The deregulation of these pathways has been reported in conditions that lead to cardiac dysfunction [31].

### 2.1. Developmental Changes and the Fetal Metabolic Switch

The human heart starts pumping blood in the fourth week of fetal development [32]. The fetal heart relies on glucose and lactate for energetic purposes, and these metabolites are obtained from the mother via the placenta [33]. Glucose is the chosen fuel not only due to hypoxia in utero but also due to substrate availability. Low oxygen levels activate the hypoxia-inducible factor (HIF)1α, which is a transcription factor that regulates the expression of genes involved in glucose metabolism, particularly in glycolysis [32]. The higher reliance on glycolysis is reflected in the expression of specific gene isoforms of glycolytic enzymes, such as hexokinase I, pyruvate kinase 4, fetal phosphofructokinase (PFK), and lactate dehydrogenase (LDH) isoform LDHA [34]. The uptake of glucose is assured by GLUT1, whereas in the adult heart, GLUT4 is the most represented isoform [31] (Figure 2). The cardiac expression of hexokinase I, HIF, and LDHA has been reported to be uniform during the late first and early second trimester in human fetuses. This glycolytic environment supports the biosynthesis of biomolecules, such as lipids and nucleotides, sustaining fetal development [35]. In utero, the heart also seems very dependent on BCAA degradation compared to that of newborns, given the significant overexpression of 17 genes associated with BCAA metabolism [33]. The expression of genes from fatty acid metabolism (e.g., fatty acid binding protein 4 (FABP4), FABP2, and retinol-binding protein (RBP)) was reported to increase as the heart matures [35]. The energetic metabolism changes, from glycolysis to FAO, during the first weeks after birth (Figure 1). The upregulation of CPT1A transcripts suggests an elevation in medium and long-chain FA oxidation in the newborn heart [33]. The increased levels of oxygen and decreased activity of HIF1α are key determinants for this metabolic remodeling of the myocardium [34].

The metabolic switch towards OXPHOS is dependent on the mitochondrial oxidative capacity, which increases in mature and terminally differentiated cardiomyocytes. In humans, cardiomyocytes proliferate during the first months of the postnatal life, after which these cells are only capable of hypertrophic growth (Figure 1). Mitochondria are small and round in the fetus, but in the neonatal heart, mitochondria fuse, increasing in size, and the cristae become denser. Increased biogenesis, together with the overexpression of peroxisome proliferator-activated receptor-gamma coactivator (PGC)-1α, occurs in the early phases of postnatal life, doubling the mitochondrial mass [34,36] (Figure 1). PGC-1β is also essential for mitochondrial maturation and dynamics during postnatal development. Both PGC-1α and PGC-1β regulate the expression of genes involved in fusion and fission processes [37]. The downregulation of HIF1α is also essential for mitochondrial maturation and, consequently, for increasing OXPHOS activity [38] (Figure 1).

### 2.2. Metabolic Changes in the Injured Heart

In the pathological remodeling of the heart, metabolic changes occur to compensate for impaired fuel utilization and loss of mitochondrial functionality that may occur at advanced stages. This remodeling is characterized by a shift in the energetic fuel, from FFAs to glucose (towards a fetal phenotype), with impact on cardiomyocytes function [12]. Nevertheless, there are discrepancies in the literature regarding the energetic choices of the failing heart, which seems to depend on the animal model, duration of cardiac disease, and on the availability of energetic substrates [11].

Ischemic heart is more reliant on glycolysis due to an oxygen deficit; however, the glycolysis rate is not enough to cope with ATP decline. Increased glucose uptake and glycolysis seems to be regulated by AMPK and not by insulin-dependent phosphoinositide 3-kinase (PI3K) signaling. AMPK phosphorylates and activates PFK2 during ischemia, leading to increased production of fructose-2,6-biphosphate and, consequently, to the activation of PFK1 [39]. AMPK-dependent mechanisms are also implicated in the translocation of GLUT4 to the cell membrane during ischemia, increasing glucose uptake [26]. However, increased glycolysis becomes uncoupled from pyruvate oxidation. The decrease in mitochondrial pyruvate metabolism seems to be due, at least in part, to the diminished activities of mitochondrial pyruvate carrier (MPC) and pyruvate dehydrogenase (PDH) [40,41]. The cytosolic pyruvate that accumulates is converted into lactate by the bidirectional enzyme LDH (Figure 2). Increased expression of LDHA, the isoform more likely to produce lactate from pyruvate, was reported in the Dahl salt-sensitive rat model of heart failure with a preserved ejection fraction (HFpEF) [40]. The relative abundance of lactate is higher than pyruvate, so lactate turns out to be the main supplier of acetyl-CoA, and lactate consumption decreases [41]. Increased lactate uptake was also reported in the heart from patients with HFrEF [25]. Monocarboxylate transporter (MCT) isoforms 1 and 4 are key lactate transporters in cardiac muscle (Figure 2), and enhanced expression of MCT4 was observed following ischemia; however, this isoform is mainly associated with lactate efflux [42]. In fact, cardiomyocytes seem to release and consume lactate at the same time [43]; endogenously produced lactate is mainly released, whereas most of the consumed lactate is oxidized, as it is an important source of energy for the heart [44]. Lactate is oxidized by the mitochondrial lactate oxidation complex (mtLOC) [40,43] (Figure 2). Impaired activity of mtLOC may occur in a failing heart, leading to intracellular lactate overload that is accompanied by enhanced H^+^ levels and a pH decrease. Subsequently, ATP is consumed by ion channels to reestablish pH, and cardiac muscle contractibility becomes impaired [40].

Decrease in fatty acid uptake and oxidation (Figure 2) was reported in late-stage heart failure due to increased phosphorylation and ubiquitination of CD36. This leads to the reduced activation of peroxisome proliferator-activated receptor alpha (PPARα; the cardiac abundant isoform of PPAR family), as FFAs are endogenous ligands of this transcription factor, and, thus, CD36 expression is limited [45]. Moreover, PGC-1α/PPARα downregulation suppresses FAO activity [12]. The decrease in CPT1 activity aggravates the lack of a fatty acyl supply for OXPHOS, and LCFA can be converted into the cardiotoxic species. The decline in CPT1 activity is related to a shift from CPT1B to CPT1A expression (the predominant fetal isoform). Short chain fatty acids (SCFAs) can overcome the lack of CPT1 activity and represent an important fuel for OXPHOS in the failing heart [46] (Figure 2). SCFAs are mainly produced by gut microbiota, with acetate being the most abundantly produced metabolite that can be used as energetic fuel and in epigenetic regulation [25,46]. Nevertheless, the downregulation of PGC1α impairs mitochondrial biogenesis and, consequently, reduces mitochondrial density and worsens the energy crisis, by being PGC1α expression fine-tuning critical for cardiac homeostasis.

In the insulin-resistant state of heart failure, glucose uptake and oxidation decrease [11]. Being a typical insulin-targeted cell, cardiomyocyte metabolism is affected by failure in insulin signaling. The increased content of circulating FFAs further inhibits insulin signaling by targeting insulin receptor substrate 1 (IRS1) and AMPK [47], reducing glucose uptake and inducing a higher reliance of cardiac metabolism on lipids for energetic purposes (approximately 90% of energy) [48]. Still, increased FAO may not be enough to compensate for FFAs uptake by cardiomyocytes, resulting in an ectopic lipid accumulation (a condition known as cardiac steatosis) of TG and other forms of lipids, such as ceramides and diacylglycerols (DAG). Lipotoxicity results in the loss of proton gradient and, therefore, in decreased ATP generation. Ultimately, the loss of mitochondrial membrane potential and the release of cytochrome c activates apoptosis, contributing to cardiomyopathy in obese and diabetic patients [22,49,50]. However, the heart has mechanisms to control lipotoxicity. For example, cardiac progesterone receptor membrane component 1 (PGRMC1) enhances mitochondrial respiration and FAO rates, preventing the accumulation of toxic lipids [50]. Increased circulating levels of BCAAs (i.e., Val, Leu, Ile) have also been related to insulin resistance through the BCAA-induced activation of the mechanistic target of rapamycin (mTOR) in the muscle and the direct toxicity of BCAA products, such as branched-chain ketoacids (BCKA) [51] (Figure 2). BCAA oxidation rates were reported to decrease and contribute with only around 1%, for ATP generation in the heart of insulin-resistant obese rats, which is accompanied by increased production of BCKA [52]. Still, the impact of BCAA on cardiac maladaptive remodeling is unclear. Other amino acids can be used as metabolic substrates. For example, glutamate uptake was reported to increase in heart failure as a response to increased circulating levels and lower rates of cardiac perfusion [25] (Figure 2). However, the heart not only consumes but also secretes amino acids—mainly glutamine and alanine—which is a way to remove amino groups from the working heart, and it is a sign of proteolysis and impaired cardiac function [25,53].

The diabetic and failing heart can be exposed to increased ketone concentrations, which are mainly produced in the liver. Increased availability is synonymous with enhanced use, with ketones becoming a major fuel of the heart (approximately 20% of energy). However, ketone oxidation does not replace that of FFAs and glucose, thus acting as an extra source of energy [9,10]. Indeed, increased reliance on ketone oxidation leads to enhanced TCA cycle activity, oxygen consumption, and, consequently, to higher cardiac work with no change in cardiac function [10]. The role of ketone bodies in heart failure goes beyond energy supply, acting as signaling molecules (e.g., βOHB inhibits histone deacetylases) and regulators of inflammation and immune cell function with an impact on heart homeostasis [54,55].

Taken together, energetic substrates are involved in the development and progression of cardiac dysfunction. Changes in the metabolic choice have an impact on energy generation and other regulatory functions, and, as a result, on cardiac remodeling and functionality.

## 3. Metabolic Control of Stem Cells Pluripotency with Implications for Cardiac Regeneration

There is a growing interest in understanding the mechanisms of ESCs and iPSCs self-renewal and differentiation, given their potential application for cell therapy, including heart regeneration [56]. Metabolites play many roles beyond serving as substrates for energy generation and anabolic growth, such contributing to intracellular redox balance and activation of intracellular signaling cascades [14]. Moreover, several metabolites can directly regulate histone marks by serving as substrates or cofactors for chromatin-modifying enzymes. For instance, acetylation and demethylation of histones relies on the availability of the TCA cycle intermediates, citrate-derived acetyl-CoA and α-ketoglutarate [14], coupling mitochondrial metabolism to transcriptional regulation.

The energetic metabolism is a key regulator of ESCs and iPSCs self-renewal, pluripotency, and differentiation (for a review, see [13,57,58]). In rodents, naïve and primed ESCs display key differences, including signaling requirements for self-renewal and central carbon metabolism [58,59]. In particular, naïve ESCs use more OXPHOS, whereas primed ESCs rely almost entirely on glycolysis [60,61]. In contrast to a mouse, human naïve ESCs exhibit increased glycolysis compared to primed ESCs [62], reflecting species-specific differences, such as dependence on FGF signaling [13]. The adoption of a predominant glycolytic metabolism, highly dependent on glucose and glutamine, irrespective of oxygen, is shared by transformed cancer cells and is known as the Warburg effect [63], which is a cancer hallmark with relevance for therapy [64,65,66,67]. The major function of aerobic glycolysis seems to be the provision of glycolytic intermediates for anabolic reactions required during proliferation [64,68], highlighting the intricate relationship between proliferation and bioenergetics.

Mitochondria are essential regulators of stem cell function [69]. Reactive oxygen species (ROS), a secondary product of mitochondrial respiration, regulate the transcription and proliferation of stem cells [69]. Despite the dominant glycolytic metabolism, mouse primed ESCs can engage in the oxidation of substrates in the mitochondria (as glutamine) [70,71]. Naïve and primed mouse ESCs present significant differences in the mitochondria dynamics, with naïve ESCs exhibiting fragmented, perinuclear mitochondrial morphology with underdeveloped cristae, whereas primed ESCs mitochondria appear elongated, with relatively more developed cristae [72]. The predominant elongated mitochondrial network of mouse primed ESCs is not accompanied by an expected reliance on mitochondrial respiration, suggesting other regulatory roles, other than bioenergetics, for mitochondria. As for ESCs, the reprogramming of somatic cells into iPSCs is accompanied by a shift from OXPHOS to glycolysis [57,73]. In iPSC reprogramming, an early fragmentation of mitochondria governed by Drp1 occurs, and the reprogramming-induced mitochondria fission is required for the full activation of pluripotency [74].

Apart from energy, intracellular FAs and acetyl-CoA pools regulate stem cell self-renewal and differentiation by epigenetic modulation [13,14,75]. Acetyl-CoA-driven histone H3 acetylation, together with H3K4me3, results in an open euchromatin state that promotes ESCs pluripotency and self-renewal [76,77]. De novo FA synthesis is critical for stem cell pluripotency and cellular reprogramming by promoting mitochondrial fission in an ACC1-dependent manner [78]. The balance between exogenous lipids and de novo lipogenesis is key for the metabolic programming of epigenetics [79,80]. Lipid free culture conditions shift human stem cells towards a naïve to primed intermediate state, resulting in markedly de novo lipogenesis and endogenous ERK inhibition. Increased lipogenesis raises acetyl-CoA and α-ketoglutarate levels, promoting histone acetylation and DNA hypomethylation [79]. ESCs’ and iPSCs’ exits from pluripotency are generally accompanied by a metabolic switch from glycolysis to OXPHOS-dependency [62,81,82]. High glycolytic fluxes contribute to elevated acetyl-CoA pools, which drive a highly-acetylated, open chromatin landscape in proliferating stem cells, in deep contrast to differentiated cells that exhibit compacted chromatin [77,81].

Most adult endogenous progenitor/stem cells reside quiescent in hypoxic niches, unless activated by stress or injury, and have an intrinsically glycolytic mode of metabolism [13,83], minimizing ROS production and entry into proliferation. Several reports have highlighted the importance of glucose and lipid metabolism in adult stem and progenitor cells including skeletal muscle stem cells (MuSCs) (for a review, see [13,16]). Glucose is dispensable for mitochondrial respiration in proliferating MuSCs, thus becoming available for acetyl-Co-dependent histone acetylation and chromatin accessibility of genes that must be silenced upon differentiation [84]. Conversely, quiescent and differentiating MuSCs increase glucose oxidation in the mitochondria, with reduction in histone acetylation. FAO usage is also necessary for preserving MuSCs’ self-renewal, where SIRT1 deacetylates and activates PGC-1α, promoting FAO and repressing glycolysis [85]. During the exit from quiescence, MuSCs deactivate FAO in favor of glucose catabolism, decreasing NAD+, SIRT1, and its histone H4K16 deacetylation activity, leading to the activation of a myogenic transcription program and differentiation [86]. Moreover, lipid availability can also determine skeletal progenitor cells’ potential of chondrogenesis over osteogenic differentiation [87]. When lipids are scarce, skeletal progenitors activate FOXO that suppress FAO in a SOX9-depedent manner, adapting cells to an avascular life [87].

In conclusion, the balance between intrinsic metabolic needs and extrinsic metabolic constraints regulates stem cell metabolism and self-renewal, highlighting the importance of nutritional systemic factors in modulating pluripotency during development and regeneration.

## 4. Metabolic Reprogramming to Enhance Cardiac Regenerative Strategies

**Figure 3:**
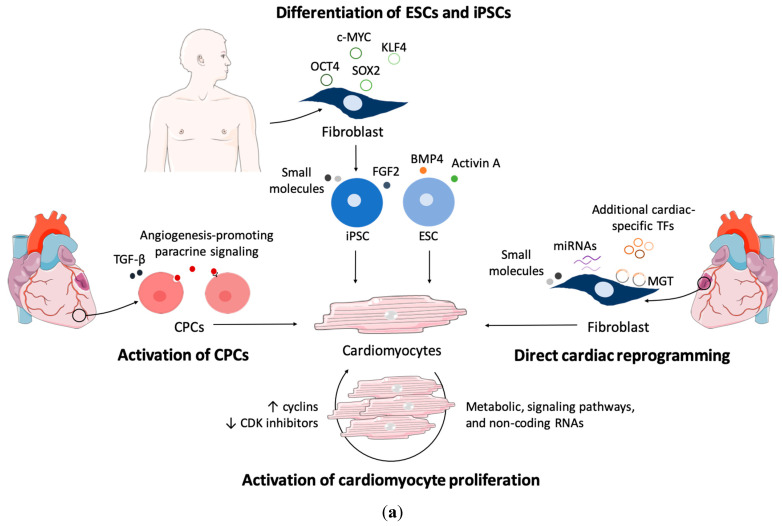

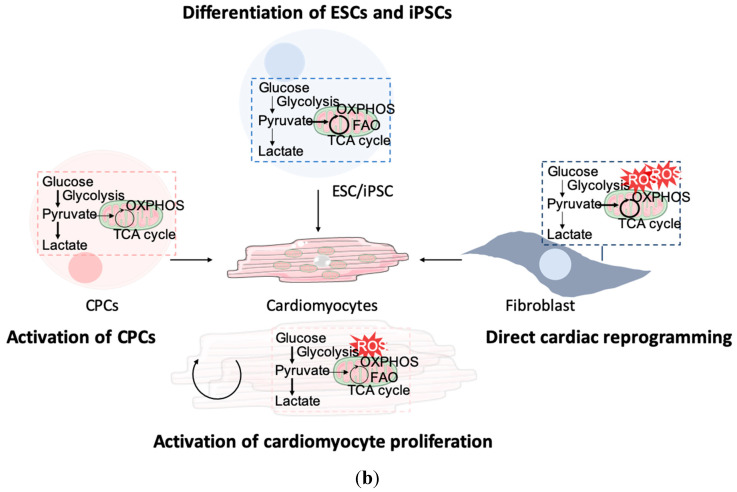
Heart regeneration strategies and metabolic manipulations to increase cardiac regeneration. (**a**) Approaches for replacing lost cardiomyocytes. By modulating factors that either promote (overexpression of cyclins) or repress (silencing of CDK inhibitors) cell cycle activity, adult cardiomyocytes, can be forced to proliferate. Metabolic and signaling pathways, as well as the expression of non-coding RNAs, can also be modulated to induce cardiomyocyte proliferation. Stimulating the differentiation of CPCs into cardiomyocytes can be achieved by treatment with TGF-β or paracrine signaling, for example. ESCs and iPSCs have been used to generate cardiomyocytes, which can be transplanted to replace cells that were lost upon cardiac injury. Direct cardiac reprogramming can be achieved by overexpressing the cardiac factors Mef2c, Gata4, and Tbx5 (termed MGT) into resident fibroblasts. The addition of additional transcription factors and small molecules, and manipulation of miRNAs, can improve reprograming efficiency. (**b**) Metabolic reprogramming to increase cardiac regenerative potential. The stimulation of glycolysis, inhibition of FAO, and decrease in oxygen increase cardiomyocyte proliferation. Activation of CPCs can be induced with high concentrations of glucose and glutamine in the media, hypoxia, and reduction in ROS levels. Differentiation of ESCs and iPSCs into cardiomyocytes can be improved by increasing FAO, decreasing glucose, and increasing exogenous lipids and galactose. For direct cardiac reprogramming, stimulation of OXPHOS and inhibition of glycolysis increases the efficiency of the process. Abbreviations: CPCs, cardiac progenitor cells; ESCs, embryonic stem cells; FAO, fatty acid oxidation; iPSCs, induced pluripotent stem cells; OXPHOS, oxidative phosphorylation; ROS, reactive oxygen species; TCA, Tricarboxylic acid cycle; up arrow: increase; low arrow: decrease.

In order to minimize the distressing outcomes of heart failure, several regenerative strategies have been proposed (Figure 3a). Current protocols of cardiomyocyte regeneration have been developed based on activating the embryonic cardiomyogenesis-induced signaling pathways and gene regulatory networks. Most studies of cardiomyocyte regeneration are focusing on the contributions of transcriptional mechanisms, including gene programming, epigenetic chromatin modifications, and biochemical differentiation cues (for review, see [88,89]). Energy metabolism is central to mammalian heart development and function, and metabolic processes can be modulated to support the contractile apparatus of regenerated cardiomyocytes. Moreover, metabolism impacts the ability of stem cell self-renewal, differentiation, and cell fate decision. Although the coordination of genetic networks with developmental bioenergetics is critical to cardiomyocyte specification, the underlying metabolic mechanisms that drive cardiac induction and differentiation, in the context of heart regeneration, are only just beginning to be appreciated (summarized in Table 1 and Figure 3b).

### 4.1. Activation of Cardiomyocyte Proliferation

The re-entry of cardiomyocytes into the cell cycle has become one of the employed strategies to regenerate cardiac tissue (for review, see [90,91]) (Figure 3a). Indeed, fetal, neonatal, and adult hearts exhibit very specific expression patterns of cardiomyocyte proliferation regulators, including gene expression, cell cycle regulators, metabolic and signaling pathways, extracellular matrix proteins and growth factors. As expected, manipulating these factors has become a strategy to force adult cardiomyocytes to re-enter the cell cycle [90,91]. Several studies have focused on the overexpression of cyclins to force mammalian (particularly mouse) cardiomyocytes to re-enter the cell cycle. For example, overexpression of cyclins D1, D2, and A2 were shown to induce cardiomyocyte proliferation, upon and in the absence of injury [92,93,94,95]. Similarly, the inhibition of cyclin-dependent kinase inhibitors, such as p21, p27, and p57, have also been shown to promote cardiomyocyte proliferation [96]. The Hippo signaling pathway has been extensively studied in cardiomyocyte proliferation, and the modulation of Hippo pathway components has been shown to promote cardiomyocyte proliferation. Indeed, activation of YAP or the depletion of Hippo or Salvador all exhibit promising results in pushing cardiomyocytes to re-enter the cell cycle [97,98]. Other signaling pathways that stimulate cardiomyocytes to proliferate and induce cardiac regeneration upon injury include the neuregulin/ERBB2/ERBB4, PI3K/AKT/CDK7, Wnt/β-catenin, PDGFR-β, and Notch signaling pathways [91,92]. Non-coding RNAs, such as microRNAs (miRNAs) and long non-coding RNAs (lncRNAs), have gained special attention over the last few years, given their potential to regulate gene expression at various levels, revealing very particular spatiotemporal profiles. In fact, several miRNAs, such as miR-128, and lncRNAs, namely *CAREL*, have been associated with cardiomyocyte proliferation and cardiac regeneration (for review, see [99,100,101]).

Another important aspect to be considered for cardiomyocyte proliferation is the fetal metabolic switch (Figure 1 and Figure 3b). Shortly after birth, mammalian cardiomyocytes lose proliferative and regenerative capacities, highlighting a connection between metabolism and cardiac differentiation. The consequence of increased exposure to oxygen leads to an intensification of OXPHOS and increased levels of ROS [20], contributing to postnatal cardiomyocyte cell cycle arrest, oxidative DNA damage, and the activation of DNA damage response [102,103]. FA utilization by the mitochondria induces a significant increase in ROS at the chromatin level compared to other nuclear compartments, highlighting chromatin as the main target of the prooxidant effect of FA utilization by the mitochondria [103]. Puente et al. investigated the influence of both hyperoxia and hypoxia in neonatal mice and reported that, while a high oxygen environment led to cardiomyocyte cell cycle arrest, lower oxygen extended the regenerative window [102]. Moreover, hypoxia was also shown to inhibit OXPHOS and reactivate mitosis in cardiomyocytes [104,105]. To extend the neonatal regenerative window, the ROS levels can be decreased, by either targeting one of its sources—for example, succinate accumulation (with the use of malonate)—or through the administration of ROS scavengers, such as N-acetylcysteine (NAC) [102,106] (Table 1). The decrease in ROS has also been shown to stimulate proliferation in adult cardiomyocytes [106].

During regeneration, cardiomyocytes, located in the border zones of the injured area, switch back to glycolytic metabolism [107]. Stimulation of glycolysis by overexpressing Glut1 leads to increased glucose uptake, which is sufficient to enhance the neonatal regenerative capacity [108]. Similarly, the deletion of PDK4, a PDH inhibitor whose normal expression is upregulated at P7, enhances cardiomyocyte proliferation and heart regeneration after myocardial infarction [109]. Inhibition of FAO also offers some promising results. Cardoso et al. demonstrated that the consumption of FA-deficient milk expanded the regenerative window up until post-natal day 21, yet this does not apply to 10-week-old mice [109]. Inhibition of CPT1 with etomoxir leads to decreased FAO and the proliferation of neonatal mouse/rat cardiomyocyte proliferation [110,111]. However, this effect does not appear to extend to adult cardiomyocytes, and etomoxir usage for extended periods of time can lead to hepatic steatosis and glucose intolerance [112,113]. Ordoño et al. have demonstrated that lactate induces changes in gene expression, which leads to cardiomyocyte proliferation. Additionally, lactate can have an impact on the surrounding environment after injury, reducing the secretion of inflammatory cytokines by cardiac fibroblasts and promoting a pro-regenerative environment for cardiomyocytes [114]. With these findings, it becomes clear that mimicking the fetal metabolic environment represents a promising strategy to activate cardiomyocyte proliferation and to increase regeneration. However, most of the studies report positive outcomes on neonatal cardiomyocytes only, and further evidence from adult hearts is needed.

### 4.2. Recruitment of Cardiac Stem or Progenitor Cells

In the normal heart, the replenishment of the cardiomyocyte population is mostly made through the proliferation of existing cardiomyocytes [115,116]. However, after injury, the recruitment and differentiation of endogenous progenitor cells have gained a more prominent role [116]. Cardiac progenitor cells (CPCs) are multipotent cells that can differentiate into both cardiomyocytes and non-myocyte cells in the heart [117] (Figure 3a). Upon activation during tissue regeneration, CPCs contribute to a newly generated pool of both cardiomyocytes and endothelial cells [118]. Reactivating pathways that are involved in cardiac lineage specification can provide a potential means to attenuate the loss of cardiomyocytes after injury [119]. In vitro differentiation of CPCs into cardiomyocytes has been demonstrated upon treatment with TGF-β. Further, paracrine signaling appears to play an important role in CPC-mediated cardiac regeneration. Smits and colleagues demonstrated that the transplantation of CPCs into the hearts of mice, after myocardial infarction, improved cardiac function [120,121]. Interestingly, the improved cardiac function, observed after CPC transplantation, has been attributed to paracrine signaling promoting angiogenesis. Enhancing angiogenesis by CPCs has been shown to promote cardiac regeneration by reducing infarcted myocardium [122,123].

CPCs niches were found in the adult mammalian heart, with these cells relying mostly on glycolytic metabolism in a low oxygen microenvironment [107] (Figure 3b). Hypoxia has been shown to increase CPCs migration in vitro and recruitment in vivo in mice, and to induce HIF-1α, which stimulates the activation of CPCs [124,125] (Table 1). The proliferation of CPCs is also important in the recruitment process and seems to be dependent on glucose and glutamine, with the latter also shown to prevent cell death induced by oxidative stress [126,127]. However, other metabolic substrates, such as lactate and pyruvate, as well as hypoxia, do not show a clear impact on CPC numbers [126,127]. The use of the antioxidant ascorbic acid has been reported to increase CPCs proliferation [128], although the impact of ROS in CPCs recruitment needs further clarification. Ultimately, CPCs recruitment seems a very promising, but under researched, strategy, and further studies are warranted.

### 4.3. Delivery of De Novo Cardiomyocytes from Differentiated ESCs/iPSCs

Several protocols were developed to generate ESCs-derived cardiomyocytes (ESCs-CMs) (reviewed in [129,130]). In vitro, cardiac differentiation of human ESCs initially involved the formation of embryoid bodies, whose cells spontaneously differentiate into derivatives of all three germ layers. This type of differentiation results in beating areas within the embryoid body, which correspond to cardiomyocytes [131,132]. Several studies have shown that ESCs-CMs exhibit typical cardiac gene and protein expressions patterns, develop an organized sarcomeric structure, and exhibit electrophysiological properties typical of cardiac muscles [133,134]. To avoid problems associated with a lack of reproducibility, serum-free protocols containing growth factors, such as BMP4, activin A, FGF2, Wnt agonists and antagonists, and vascular endothelial growth factor, were developed [130] (Figure 3a). Embryoid bodies exhibit diffusional barriers that prevent the penetration of extracellular factors [130]. As such, monolayer differentiations of human ESCs into cardiomyocytes were developed, and they aimed at being more controlled and reproducible. In a study carried out by Laflamme and colleagues, H7 human ESCs were cultured on Matrigel, in the presence of mouse embryonic fibroblast conditioned media, and subsequently differentiated upon sequential treatment, with activin A and BMP4, in serum-free conditions [135]. An alternative monolayer approach requires the use of extracellular matrix proteins, which are critical players in development and can complement responses to soluble cytokines [136]. Inductive co-cultures have also been described in cardiac differentiation protocols. Co-culture of human ESCs with mouse cell line END-2 (with features of visceral endoderm) resulted in beating cardiomyocytes, which is in agreement with the role of visceral endoderm in the differentiation of cardiogenic precursor cells in the adjacent mesoderm in developing embryos [137,138].

Despite their potential, there are a several concerns regarding the use of ESCs in research and clinics: namely, immune rejection and ethical issues [139]. Mouse and human fibroblasts can be successfully converted into iPSCs by the overexpression of pluripotency-related transcription factors Oct4, Sox2, Klf4, and c-Myc [139,140] (Figure 3a). As iPSCs are generated from cells derived from patients’ own body, iPSCs-derived cardiomyocytes (iPSCs-CM) are less prone to immune rejection, and there are no ethical concerns involved [141]. Under in vitro conditions, iPSCs can be pushed towards a cardiac fate the same way as ESCs, either through the formation of embryoid bodies or monolayer culture [141]. The use of small molecules has also been extensively exploited to increase the efficiency of cardiac differentiation. For instance, inhibiting the TGF-β [142] or activating the Wnt/β-catenin [143] signaling pathways has proven to improve this process. Furthermore, small molecules, alone, such as CHIR99021 and Wnt-C59, have been used to differentiate iPSCs into cardiomyocytes [144].

Mouse and human ESCs-CMs have higher mitochondrial OXPHOS, and inhibition causes reduction and abnormal translocation of mitochondria, which results in impaired sarcomere organization and a reduced beating rate [145]. In ESCs-CMs, glucose inhibits maturation through the pentose phosphate pathway, and permanent hyperglycemic culture conditions suppress mesoderm and cardiac transcription genes and induce poor cardiomyocyte contractility [146] (Figure 3b and Table 1). The usage of galactose and FAs instead of glucose promoted a fast maturation of human ESCs-CMs, presenting higher oxidative metabolism and improved contractile capacity [147].

Despite their advantages, the physiological immaturity of iPSCs-CMs limits their utility for cardiac regeneration [148,149]. As the commonly used RPMI/B27-based media has high glucose and low oxidative substrate levels, iPSCs-CMs fail to effectively activate FAO, and lipid availability in cultures became limited during differentiation [150]. A maturation media (MM) capable of providing oxidative substrates adjusted to the metabolic demand of human iPSCs-CMs was recently proposed [151]. MM could enhance FAO, which is reinforced with a supplementation of FAs, and exacerbated electrophysiological and mechanical maturation features [151]. Exogenous supplementation of lipids, such as palmitate, improved cardiomyocyte morphology, mitochondrial function, and promoted increased FAO in human iPSCs-derivatives [150,152]. Human iPSCs-CMs present a substantial increase in the mitochondria number and activity during the differentiation process [153]. As for ESCs-CMs, glucose exclusion is necessary to induce iPSCs-CMs maturation and the switch from glycolysis to OXPHOS [154]. BCAAs can stimulate mTOR activation, which promotes metabolic reprogramming to glycolysis from FAO through HIF-1α [155]. Both the inhibition of mTOR and activation of AMPK by AICAR promote human iPSCs-CMs maturation via p53-induced quiescence [156,157], suggesting that downstream pathways of BCAAs can regulate cardiomyocyte maturation.

Together, these findings indicate that ESCs and iPSCs-CMs immaturity can be overcome by the modulation of the intracellular metabolism driving cardiomyocyte differentiation, promoting FAO, improvement of mitochondrial function, and reducing glucose metabolism.

### 4.4. Direct Reprogramming of Fibroblasts into Induced Cardiomyocytes

Most of the human heart is composed of cardiac fibroblasts, accounting for more than 50% of all the cells, and upon cardiac injury, these cells play an important role in scar formation [158]. Therefore, the substantial population of cardiac fibroblasts has emerged as a potential source of cardiomyocytes for regenerative medicine. Cardiomyocytes can be generated directly from fibroblasts without having to pass through a pluripotent state, giving rise to induced cardiomyocytes (iCMs) [159] (Figure 3a). This type of cell reprogramming—direct cell reprogramming, or transdifferentiation—offers some advantages in relation to iPSC-based differentiation, in the sense that iPSC generation is a time-consuming process with risk of tumor formation from residual undifferentiated cells [160]. A pioneer study, in 2010, identified the cardiomyocyte-inducing factors Gata4, Mef2c, Tbx5 (termed GMT), which, upon overexpression in cardiac fibroblasts, isolated from αMHC (myosin heavy chain) promoter-driven EGFP-IRES-puromycin transgenic mice (αMHC-GFP), led to an increased expression of cardiac markers: namely, αMHC (reported by GFP expression), cardiac troponin T (cTnT), and α-actinin [159]. Furthermore, iCMs resulting from the transduction of fibroblasts with GMT presented epigenetic alterations and genetic expression patterns similar to those of postnatal cardiomyocytes and spontaneous contraction, as evidenced by intracellular Ca^2+^ flux. Importantly, the possibility that these iCMs were derived either from a subpopulation of stem-like cells or cardiac progenitors was excluded, as GMT-transduced tail-tip dermal fibroblasts also exhibited the same characteristics as cardiac fibroblasts [159]. In vivo cardiac reprogramming has been achieved, with the in situ delivery of retroviruses expressing GMT, after myocardial infarction in the mouse, leading to a conversion of 15% of cardiac fibroblasts into iCMs, decreasing scar size and improving cardiac function [161].

In order to enhance reprogramming efficiency, many subsequent studies aimed at testing additional transcription factors. The addition of Hand2 (GHMT), for instance, was shown to improve cardiac conversion efficiency [162]. Additionally, 5-factor (GHMT + Nkx2.5) [163] and 7-factor (GMT + Myocd + Srf + Mesp1 + Smarcd3) cocktails were shown to further enhance cardiac reprogramming [164]. Chemical generation of iCMs has been made possible due to the use of small molecules, which function, mainly, by regulating specific signaling pathways, as well as epigenetic and metabolic processes (reviewed in [165]). Indeed, human fibroblasts were also subjected to direct reprogramming studies, although GMT transduction proved to be insufficient to induce cardiac conversion, and additional factors were simultaneously required [166,167]. Many protocols depend on retroviral and lentiviral vectors for gene delivery. Because they integrate into the host genome, endogenous gene expression destabilization and insertional mutagenesis could hinder clinical application [168]. Non-integrative viruses, such as Sendai virus, expressing cardiac reprogramming factors have been shown to reduce fibrosis after myocardial infarction and improve cardiac function [169].

Cardiac reprogramming factors function in a combinatorial way by co-occupying regulatory elements, thus inducing the activation of cardiac-specific gene expression [170]. In fact, each transcription factor plays different roles by activating cardiac-specific enhancers, with Mef2c playing a key role in initializing this process, compared to Gata4 and Tbx5. The balance and stoichiometry between transcription factors play an important role in cardiac fate commitment. The transduction of polycistronic MGT into fibroblasts resulted in the expression of Mef2c at relatively high levels and Gata4 and Tbx5 at low levels, which promoted reprogramming efficiency [171]. Owing to several intrinsic barriers, direct cardiac reprogramming is still a very inefficient process. For fibroblasts to be directly reprogrammed, significant epigenetic alterations must occur in order to repress fibroblast identity and activate the cardiomyocyte program [172,173]. Another important issue is the heterogeneity and asynchronous nature of the reprogramming process. Using single-cell RNA sequencing, Liu and colleagues discovered molecularly distinct subpopulations of cells during the reprogramming process, and they further identified *Ptbp1* as a barrier of direct cardiac conversion [174]. Another reprogramming barrier worth noting is aging [175], as age-related inflammation, and possibly senescence, can hamper direct cardiac reprogramming [176].

Fibroblasts are metabolically different from cardiomyocytes, and during direct cardiac reprogramming, a shift from glycolysis to OXPHOS has been reported [174] (Figure 3b). Therefore, manipulating fibroblast metabolism to resemble that of cardiomyocytes seems to be a good strategy to improve direct cardiac reprogramming efficiency (Table 1). Fibroblasts with downregulation of HIF-1α, an important regulator of glycolysis, are more successfully reprogrammed [177]. Conversely, the use of rotenone, an inhibitor of mitochondria respiration, and knockdown of the TCA cycle enzyme isocitrate dehydrogenase 3α (IDH3A) decreases reprograming efficiency, while IDH3A upregulation led to increased reprogramming [178]. High ROS levels can also affect the efficiency of the process, and the use of antioxidants could boost this process. Selenium and ascorbic acid (vitamin C) supplementation have been shown to enhance reprogramming efficiency, in vitro, by increasing cardiac gene expression [128,179], and vitamin E nicotinate promotes heart damage repair through reprogramming in vivo [180]. However, ROS signaling is required for direct reprograming, so a delicate balance of ROS levels seems imperative for the success of direct cardiac conversion [181]. The specific culture conditions can also impact transdifferentiation of fibroblasts in vitro, which may not be translated into significant improvements in vivo. Differential culture methods and reagents, as fetal bovine serum, have been implied in the inefficiency of this process, and serum-free conditions were reported to improve transdifferentiation [182,183].

### 4.5. Systemic Metabolic Strategies for Heart Regeneration

#### 4.5.1. Nutrient Signaling

A variety of caloric and dietary restriction regimens have been demonstrated to improve overall health and promote tissue regeneration [184]. Additionally, supplementation of metformin, resveratrol, and certain nutrients have been shown to favor cardiac tissue regeneration and remodeling in rodents. Nutritional and pharmacological modulators (e.g., vitamin E, ascorbic acid, selenium, taurine, and tocopherol) also promote mild to moderate cardioprotective effects after reperfusion injury [185,186], although substantial limitations, such as fast clearance, limit its activity. Metformin acts, both directly and indirectly, through the activation of AMPK and inhibition of mTORC1 and by improving mitophagy and overall mitochondrial homeostasis [187]. A postconditioning effect for metformin in experimental ischemia has been described (Table 1). Metformin maintained both cell viability and membrane stability of rat H9C2 cardiomyoblast cells after induced ischemic injury by alleviating the decrease in nitrate levels [188]. The increased levels of nitrate provided by metformin led to nitrate oxidative species (NOS)-independent nitrate oxide (NO) production and decrease in intracellular oxidative damage after ischemic cell injury [188]. A cardioprotective role for resveratrol was reported in a mouse model of HFpEF [189]. Resveratrol activates SIRT1, which prevents cardiac fibrosis and ROS production in cardiomyocytes [190]. Excessive myocardial ROS has a crucial role in ejection fraction preservation, leading to collagen uncoupling and formation of oxidative stress, decreased nitric oxide bioavailability, and heart failure. Resveratrol exerts a protective action against HFpEF-induced adverse cardiac remodeling, alleviating cardiac stiffness and oxidative stress by decreasing Smad3 acetylation and transcriptional activity via SIRT1 activation [189]. Whether systemic metabolic fuels can directly impact cardiomyocytes’ cell fate specification and proliferation, as well as potentiating cardiac regeneration, are exciting possibilities that need further investigation.

#### 4.5.2. Gene Therapy

Gene therapy strategies have been used to treat age-related diseases, including heart failure where documented targeted genes are involved, directly or indirectly, in metabolic-signaling pathways. Overall, the metabolic decay with age is accompanied by disrupted mitochondrial fitness, as a result of the activity decline of the master regulators PGC-1α/β [191]. Hearts from telomerase-deficient mice present deregulation of PGC-1α/β-related gene networks, mitochondria with defective electron transport chain activity, decreased ATP production, and low expression of ROS detoxifying enzymes [191]. Enforced telomerase reverse transcriptase (Tert), PGC-1α expression or germline deletion of p53 in telomerase-deficient mice restores PGC network expression, mitochondrial respiration, cardiac function, and gluconeogenesis [191]. Moreover, aged wild-type mice, infected with AAV9-Tert, present improved mitochondrial fitness, with partial rescue of PGC-1α, ATP synthase, and ERRα expression in the heart [192], and they are protected from heart failure after myocardial infarction (MI), with restored metabolic activity in the infarcted hearts [192,193] (Table 1). Overall, telomerase activation, and its impact on metabolism remodeling, could be a therapeutic strategy to prevent heart failure after myocardial infarction [193]. Under stress conditions, SIRT1 deacetylates FOXO1/3, activating the expression of the manganese-dependent superoxide dismutase and catalase anti-oxidant enzymes, protecting the heart against oxidative stress and apoptosis in ischemia/reperfusion (I/R) injury [194,195,196]. Adenoviral-mediated overexpression of Sirt1 blunted the increase in the atrial natriuretic factor and α-skeletal actin expression, and it inhibited cell size enlargement and FAO repression of phenylephrine-stimulated neonatal rat cardiomyocytes [197]. The mitochondrial deacetylase SIRT3 can also potentiate the mitochondria to eliminate excessive ROS through deacetylation and activation of superoxide dismutase [198]. The lncRNA lncDACH1 is highly expressed in high glucose-treated or diabetic cardiomyopathy mouse hearts, and its knockdown in neonatal mouse ventricular cardiomyocytes (NMCVs) attenuates mitochondrial oxidative stress and apoptosis. Additionally, the lncDACH1 knockout mouse has enhanced cardiac function due to the prevention of SIRT3 degradation through ubiquitination-related degradation of SERCA2a [199]. Similarly, SIRT1 increases SERCA2 pump activity during heart failure by SERCA2 acetylation at lysine 492 [200]. Additional lncRNAs have been reported to modulate mitochondrial dynamics in the heart [101]. Malat1 has increased expression in cardiac tissues and isolated endothelial cells in the peri-infarct region, and cardiac microvascular endothelial cells (CMECs)-specific knockout, in vivo, leads to persistent mitochondrial dysfunction, priming hypoxia-induced endothelial cell damage, apoptosis, capillary destruction, and cardiac microvascular injuries [201]. Malat1 silencing, both in vivo and its knockdown in CMECs, results in decreased Mfn1 through the regulation of miR-26b-5p, which leads to disrupted mitochondrial dynamics and activation of apoptosis [201]. These emerging insights highlight the possibility to explore lncRNAs-mediated-metabolism as a strategy to improve cardiac regeneration and heart function [101].

LARP7 was recently shown to preserve mitochondrial homeostasis and cardiac function. When the ataxia telangiectasia mutated (ATM)-mediated DNA damage response pathway is activated, oxidative stress leads to the decline of LARP7 and compromises the homeostasis and deacetylase activity of SIRT1, which leads to disruption of mitochondrial biogenesis. The cardiac-specific NKX2.5^Cre^:LARP7^f/f^ and Myh6^Cre^:LARP7^f/f^ conditional knockout mice developed heart failure with disturbed mitochondria function and morphology. Rescue of LARP7 expression by the AAV9- mediated gene delivery remarkably had a protective role against heart failure, improving the pump function of the infarcted heart [202]. Nrf1 is considered a master regulator of redox balance and a core component of stress adaptation in the heart [203]. Overexpression of Nrf1 confers protection to I/R injury in the adult mouse heart by activating ROS scavengers, including the detoxifying enzyme Hmox1, glutathione, and NADPH metabolism, as well as increasing proteasomal activity in the heart [203].

Overall, gene targeting seems promising to rescue impaired metabolism and mitochondria function in response to cardiac injury or heart pathologies.

## 5. Conclusions

Cellular metabolism and nutrient signaling are determinant for cardiomyocyte function in the adult heart, and extensive metabolic reprogramming takes place at developmental stages and in the injured heart. In fact, a hallmark of heart failure is the metabolic remodeling, characterized by a shift in energetic fuels and pathways towards a fetal phenotype (from FAO to glycolysis), which compensate for the loss of cardiomyocyte function. Understanding the key metabolic signals that drive cardiac regeneration post-injury, in vivo, is warranted for metabolically treating the diseased heart. Due to the extended impact of metabolites in cell signaling and epigenetic remodeling, changes in the metabolic fuel impact energy generation, other regulatory functions, and, as a result, cardiac remodeling and functionality. Several cellular strategies have been designed for regenerating the adult mammalian heart, with stem cells-based therapy and direct conversion of cardiac fibroblasts showing promising results. In this context, metabolic adaptations, mainly involving fuels (glucose, FAs), energetic pathways (glycolysis, OXPHOS), and the remodeling of mitochondria function (including dynamics and ROS) can promote self-renewal, differentiation, and cell lineage conversion events that are driving cardiac regeneration (Table 1 and Figure 3b). Cellular metabolism represents the balance of intrinsic metabolic needs and extrinsic metabolic constrains. Understanding how global shifts in nutrient availability (such as lipids) translate into specific gene expression programs, controlling cellular events with relevance for cardiac regeneration, is a major area for future investigation.

## Figures and Tables

**Table 1:** Metabolic manipulations that potentiate cardiac regenerative strategies.

Cardiac Regenerative Approach	Metabolic Target	Strategy	Species	Cell Type	Impact	References
Activation of cardiomyocyte proliferation	Glycolysis	Glut 1 overexpression in vivo	Mouse	Neonatal cardiomyocytes	Increases proliferation and decreases fibrosis post-injury	[90]
	Glycolysis	PDK4 knockout in vivo	Mouse	Adult cardiomyocytes	Increases proliferation and improves heart function	[91]
	FAO	FAs -deficient milk in vivo	Mouse	Neonatal cardiomyocytes	Extends the post-natal regenerative window	[91]
	CPT1	Etomoxir supplementation in vivo	Mouse Rat	Neonatal and adult cardiomyocytes	Enhances cardiac efficiency post-injury	[92,93]
	Lactate	Supplementation in vitro	Mouse	Cardiac fibroblasts	Pro-regenerative environment for cardiomyocytes post-injury	[94]
	ROS	NAC, malonate supplementation in vivo	Mouse	Neonatal and adult cardiomyocytes	Extends the post-natal regenerative window	[95,96]
Recruitment of cardiac stem or progenitor cells	HIF-1α	Hypoxia in vivo and in vitro	Mouse	CPCs	Promotes migration and recruitment	[97,98]
	Glucose, glutamine	Supplementation in vitro	Mouse	CPCs	Increases proliferation and prevents cell death induced by oxidative stress	[99,100]
	ROS	Ascorbic acid supplementation in vitro	Mouse	CPCs	Increases proliferation	[101]
Delivery of De Novo cardiomyocytes from differentiated ESCs/iPSCs	Glucose	Low	Human	ESCs-CMs iPSCs-CMs	Physiological support for cardiac development	[102]
	Glucose	High	Mouse	ESCs-CMs	Suppresses mesoderm and cardiac transcription genes	[103]
	mTOR AMPK	Torin1 AICAR supplementation in vitro	Human	iPSCs-CMs	Cardiomyocyte maturation	[104,105]
	Galactose, FAs	Supplementation in vitro	Human	iPSCs-CMs ESCS-CMs	Improves contractile capacity and maturation	[106,107]
	FAO	MM in vitro	Human Mouse	iPSCs-CMs	Metabolic maturation	[107]
Direct reprogramming of fibroblasts into iCMs	Glycolysis	HIF-1α knockdown in vitro	Mouse	Neonatal cardiac fibroblasts	Enhances reprogramming efficiency	[108]
	OXPHOS TCA cycle	Rotenone, IDH3A knockdown in vitro	Mouse	Embryonic fibroblasts	Decreases reprogramming efficiency	[109]
	ROS	Selenium, ascorbic acid supplementation in vitro	Mouse	Embryonic, neonatal cardiac and tail tip fibroblasts	Enhances reprogramming efficiency	[101,110]
	ROS	Vitamin E nicotinate supplementation in vivo	Rat	Cardiac fibroblasts	Improves heart damage repair through reprogramming	[111]
Nutrient signaling	AMPK	Metformin supplementation in vitro	Rat	H9C2 cardiomyoblasts	Nitrate-dependent decrease in oxidative damage	[112]
	SIRT1	Resveratrol supplementation in vivo	Mouse	Neonatal cardiomyocytes	Ejection fraction preservation, decreases cardiac stiffness and oxidative stress	[113]
Gene therapy	Mitochondria	AAV9- Tert overexpression in vivo	Mouse	Adult heart	Improves mitochondrial fitness and activity, protects against heart failure after MI	[114,115,116]
	Mitochondria	Ad5-CMV-Sirt1 overexpression in vitro	Rat	Neonatal cardiomyocytes	Protects against oxidative stress, FAO inhibition and cell size enlargement	[117]
	Mitochondria	Malat1 knockdown in vitro knockout in vivo	Mouse	CMECs	Mitochondrial dysfunction, apoptosis and microvascular injuries	[118]
	Mitochondria	AAV9- LARP7 overexpression in vivo	Mouse	Adult heart	Protects against heart failure, improves pump function	[119]
	ROS	lncDACH1 knockdown in vitro knockout in vivo	Mouse	NMCVs Adult heart	SIRT3-mediated attenuation of mitochondrial oxidative stress	[120]
	ROS	AAV9-Nrf1 overexpression in vivo	Mouse	Adult heart	Protects against I/R injury by activating ROS scavengers	[121]

Abbreviations: AAV9, Adeno-associated virus serotype 9; AICAR, 5-Aminoimidazole-4-carboxamide ribonucleoside; AMP, 5′ adenosine monophosphate; AMPK, AMP-activated protein kinase; CMECs, Cardiac microvascular endothelial cells; CPCs, cardiac progenitor cells; CPT1, carnitine palmitoyltransferase 1; ESCs, embryonic stem cells; ESCs-CMs, embryonic stem cells-derived cardiomyocytes; FAs, fatty acids; FAO, fatty acid oxidation; Glut 1, Glucose transporter 1; HIF-1α, Hypoxia-inducible factor-1α; iCMs, induced cardiomyocytes; IDH3A, Isocitrate dehydrogenase 3α; iPSCs, induced pluripotent stem cells; iPSCs-CMs, induced pluripotent stem cells-derived cardiomyocytes; I/R, ischemia/reperfusion; LARP7, la ribonucleoprotein domain family member 7; MI, myocardial infarction; MM, maturation media; mTOR, mechanistic target of rapamycin; NAC, N-acetyl-cysteine; NMCVs, neonatal mouse ventricular cardiomyocytes; Nrf1, nuclear respiratory factor 1; OXPHOS, oxidative phosphorylation; PDK4, pyruvate dehydrogenase kinase 4; ROS, reactive oxygen species; Sirt, sirtuin; TCA, Tricarboxylic acid cycle; Tert, Telomerase reverse transcriptase.
